# Deconditioning in quiescent Crohn’s disease patients with heightened fatigue perception

**DOI:** 10.1093/ecco-jcc/jjae194

**Published:** 2025-01-09

**Authors:** Jordan J McGing, Sébastien Serres, Rosemary Nicholas, Ayushman Gupta, Shellie J Radford, Aline V Nixon, Joanne Mallinson, Christopher Bradley, Stephen Bawden, Susan T Francis, Paul L Greenhaff, Gordon W Moran

**Affiliations:** Sir Peter Mansfield Imaging Centre, University of Nottingham, Nottingham, United Kingdom; Faculty of Medicine and Health Sciences, University of Nottingham, Nottingham, United Kingdom; Sir Peter Mansfield Imaging Centre, University of Nottingham, Nottingham, United Kingdom; Faculty of Medicine and Health Sciences, University of Nottingham, Nottingham, United Kingdom; Faculty of Medicine and Health Sciences, University of Nottingham, Nottingham, United Kingdom; NIHR Nottingham Biomedical Research Centre, Nottingham, United Kingdom; David Greenfield Human Physiology Unit, Faculty of Medicine and Health Sciences, University of Nottingham, Nottingham, United Kingdom; David Greenfield Human Physiology Unit, Faculty of Medicine and Health Sciences, University of Nottingham, Nottingham, United Kingdom; Sir Peter Mansfield Imaging Centre, University of Nottingham, Nottingham, United Kingdom; NIHR Nottingham Biomedical Research Centre, Nottingham, United Kingdom; Sir Peter Mansfield Imaging Centre, University of Nottingham, Nottingham, United Kingdom; NIHR Nottingham Biomedical Research Centre, Nottingham, United Kingdom; MRI Physics Group, Department of Medical Physics and Clinical Engineering, Singleton Hospital, Swansea, United Kingdom; Sir Peter Mansfield Imaging Centre, University of Nottingham, Nottingham, United Kingdom; NIHR Nottingham Biomedical Research Centre, Nottingham, United Kingdom; David Greenfield Human Physiology Unit, MRC/Versus Arthritis Centre for Musculoskeletal Ageing Research, NIHR Nottingham Biomedical Research Centre, Faculty of Medicine and Health Sciences, University of Nottingham, Nottingham, United Kingdom; NIHR Nottingham Biomedical Research Centre, Nottingham, United Kingdom; Translational Medical Sciences, School of Medicine, Faculty of Medicine and Health Sciences, University of Nottingham, Nottingham, United Kingdom

## Abstract

**Background and Objective:**

Inflammatory bowel disease (IBD) fatigue aetiology is poorly understood. This study quantified body composition and physical function alongside proton magnetic resonance imaging (^1^H MRI) and spectroscopy (^31^P MRS) measures of organ structure and function in quiescent Crohn’s disease patients (CD) and healthy volunteers (HVs), to identify a physiological basis for IBD fatigue.

**Methods:**

Body composition was determined using dual-energy X-ray absorptiometry and ^1^H MRI. Knee extensor isometric strength and isokinetic fatigue were measured using dynamometry. ^1^H MRI was used to quantify cardiac output, cerebral blood flow (gmCBF), and brain oxygen extraction fraction (OEF) at rest, and during supine, steady-state exercise, and recovery. ^31^P MRS was used to quantify post-exercise muscle phosphocreatine (PCr) resynthesis.

**Results:**

Sixteen CD and 12 HV (age, sex, and BMI matched) were recruited. Fatigue perception was greater (13.9 ± 1 vs 8.3 ± 0.9, *P* = .001), and daily step count was less (5482 ± 684 vs 8168 ± 1123, *P* = .04) in CD. During steady-state exercise, gmCBF was less in CD (653 ± 30 vs 823 ± 40 mL/min, *P* = .003). Cardiac output and brain OEF were no different. Post-exercise PCr resynthesis was less in CD (17.2 ± 2.0 vs 25.3 ± 2.4 mM·min^−1^, *P* = .02). Body composition, isometric strength, and isokinetic fatigability were no different.

**Conclusions:**

CD self-reported increased fatigue perception and exhibited a slower rate of post-exercise PCr resynthesis compared to HV. This occurred independently of changes in body composition, muscle strength, and fatigability. IBD fatigue may be linked to peripheral muscle deconditioning and lower gmCBF during submaximal exercise.

## Graphical Abstract

**Graphical Abstract:**
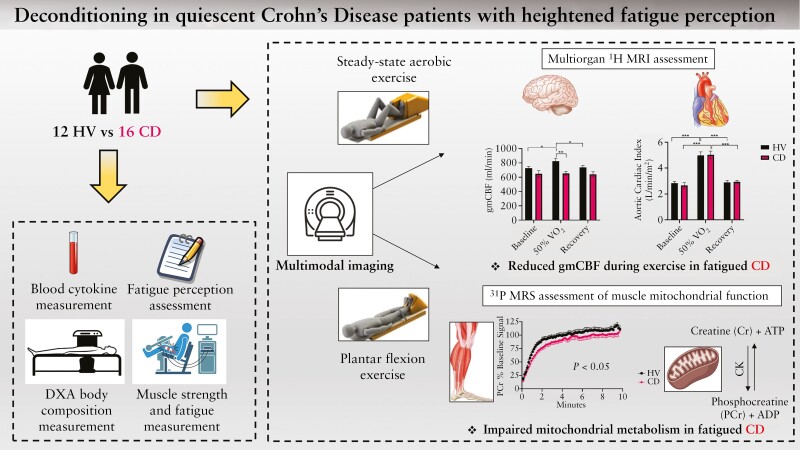

## 1. Introduction

Heightened fatigue perception is a significant clinical burden for inflammatory bowel disease (IBD) patients, reflected by overt symptoms of tiredness and lethargy during activities of daily living.^1,2^ Consistent with the association between acute inflammation and fatigue,^3–5^ the prevalence of increased fatigue perception in active IBD has been reported to be as high as 86%.^6^ Furthermore, increased fatigue perception commonly persists during disease remission in a large proportion of IBD patients, independent of known reversible clinical causes,^7^ which impedes the clinical management of fatigue symptoms. Fatigue prevalence (i.e. increased fatigue perception) is reported in 50% of patients with inactive or mild IBD, relative to 22% of healthy non-IBD volunteers,^8^ and patients report the burden of fatigue perception to be greater than that of gastrointestinal symptoms.^9^ Indeed, fatigue perception impairs quality of life sufficiently to be reported as the commonest reason for work absence in IBD.^10^ As a result, IBD fatigue has been highlighted as a research priority.^11^

The aetiology of IBD fatigue is multifactorial^12^ and is likely to originate from peripheral and central mechanisms.^13^ Premature exercise fatigue in IBD could, for example, be attributable to deficits of central motor and sensory drive and/or reduced motor unit size and recruitment.^14^ Furthermore, a decline in cardiorespiratory and cardiovascular functional capacity,^15^ and/or reduced muscle mass and metabolic quality, clinically described as deconditioning, may modulate premature exercise fatigue in IBD. Indeed, the exercise workload and rate of oxygen consumption (VO_2_) attained at the point of volitional fatigue during incremental intensity exercise in IBD patients is lower as compared to healthy volunteers (HVs).^15–17^ Furthermore, the workload at which blood lactate accumulation increases in trajectory during an incremental exercise test in IBD patients has been reported to be lower than that observed in HV,^17^ with the rate of heart rate recovery following exercise cessation being slower.^18^ In line with this evidence of physical deconditioning, performance in laboratory-based assessments designed to mimic real-world functionality, such as the sit-up and stand test, is also lower in Crohn’s disease (CD) relative to HV.^19^

This study aimed to quantify body composition, strength, and exercise fatigue during repeated muscle contractions in quiescent IBD patients and HVs to provide robust evidence of increased fatigue perception and premature exercise fatigue in IBD. Furthermore, to better understand its aetiology, this study also undertook the most comprehensive metabolic and physiological phenotyping of an IBD cohort to date using multi-organ magnetic resonance imaging (MRI) and ^31^P MR spectroscopy (MRS) approaches, which included dynamic measurements of metabolic and physiological function during and following in-bore exercise.

## 2. Materials and methods

### 2.1 Volunteer recruitment and screening

This was a single-center observational study recruiting CD participants in remission from Nottingham University Hospitals (NUH) Trust. NUH outpatients were screened from clinic lists by a research nurse and 24 eligible patients were contacted. Age, sex, and BMI-matched HVs were recruited through advertisements on NUH Trust campuses as non-CD controls. The study conformed to the Declaration of Helsinki and was approved by the East Midlands Nottingham 1 Research Ethics Committee (17/EM/0431), and the protocol was registered on ClinicalTrials.gov (NCT03670693).

Primary endpoints comprised measurement of post-exercise skeletal muscle phosphocreatine (PCr) resynthesis rate, cardiac output, and cerebral blood flow and fractional oxygen extraction at rest before, and during and after supine steady-state exercise. Secondary endpoints included supine peak VO_2_, isometric knee extensor strength, work output during repeated isokinetic knee extension maneuvers, whole body fat and lean mass, hospital anxiety and depression questionnaire scores, 7-day pedometer data, IBD fatigue scale (IBDF) scores, and quality of life measures through the Crohn’s and ulcerative colitis questionnaire-32 (CUCQ-32).

Sample size estimations were performed for the primary endpoint of skeletal muscle PCr recovery rate. Post-exercise PCr ½ time is 35 seconds ± 3 in HVs vs 45 ± 4 seconds in COPD patients who present with premature exercise fatigue.^20^ Assuming a power of 80% and α = 0.05, 4 subjects in each group would be required to show a difference in phosphorus magnetic resonance spectroscopy (^31^P MRS)-derived PCr recovery kinetics between an HV group and a fatigable group with chronic disease.

CD patients (age 16-75 years, BMI < 30 kg/m^2^) in remission were recruited to the study. Inclusion criteria were disease remission, defined as an absence of visible intestinal inflammation on recent ileocolonoscopy or cross-sectional imaging performed within 12 weeks of inclusion to this study, Harvey Bradshaw index < 4, and C-reactive protein (CRP) <5 mg/dL or a fecal calprotectin of < 250 μg/g. Age and sex-matched HVs with no chronic health conditions were also recruited.

All study participants completed a health screening visit. This included an electrocardiogram, a full blood count, blood clinical chemistry, liver function estimation, electronic glomerular filtration rate estimation, CRP, and vitamin D (NUH Trust Clinical Biochemistry department). Serum blood was obtained and analyzed in-house using enzyme-linked immunosorbent assay kits to measure the serum concentration of Tumor necrosis factor alpha (TNFɑ), Interleukin-1 (IL-1) and Interleukin-6 (IL-6) (R&D Systems). With regard to fatigue perception, this study recruited all comers irrespective of their fatigue status. Fatigue perception was estimated using the General^6,21,22^ and Physical^23^ domains of the Multidimensional Fatigue Inventory–20 scale and the IBD fatigue scale (CD group only). Both General and Physical fatigue perception scores were used to assess heightened fatigue perception in CD by comparison to HVs using an unpaired *t*-test. Participants completed the Hospital Anxiety and Depression Scale and Montreal Cognitive Assessment. Exclusion criteria included anemia, significant electrolyte, trace element or vitamin deficiency, renal failure, hypokalemia, arthritis, or arthralgia, significant cardiovascular or respiratory disease, neurological or cognitive impairment, significant physical disability, active or previous prescriptions of corticosteroids (previous 12 weeks), surgical intervention in the last 12 weeks, and pregnancy or childbearing in the previous 6 months. We actively excluded study participants who undertook structured exercise training to control for the potential confounding effects of chronic exercise training on study endpoints.

Following screening and entry into the study, continuous physical activity tracking was undertaken for 7 days using a pedometer (OMRON HJ-321-E, OMRON Healthcare).

### 2.2 Physiology laboratory visit: body composition, and muscle and cardiorespiratory function

Body composition was assessed via dual-energy X-ray absorptiometry (DEXA, Lunar Prodigy, GE Healthcare) to determine the whole body and regional lean and fat masses. Knee extensor isometric strength was measured (Cybex Norm) by participants performing 3 maximum voluntary contractions interspersed with 60-second recovery.^24^ Work output was measured during 20 consecutive maximal isokinetic knee extensions at 90^o^/second angular velocity to maximize recruitment of all motor units.

Following a dedicated familiarization protocol, volunteers performed an incremental, supine cardiorespiratory exercise test on an air-braked Cardio Step MRI compatible ergometer (Ergospect GmbH) to determine minute ventilation (VE), carbon dioxide production (VCO_2_), VO_2_, and heart rate using an online breath by breath gas analysis system (COSMED Quark CPET, Rome, Italy). Exercise commenced at 50 W and increased by 20 W at 3-minute intervals. The test was terminated when volunteers were unable to maintain the required power output, step frequency, or when a plateau in VO_2_ was observed. Following 90 minutes of rest, a truncated supine exercise test was performed to confirm the VO_2_ peak. Exercise commenced at 50 W for 3 minutes followed by an immediate increase to the peak workload achieved during the previous initial incremental test. The test was continued until volitional exhaustion using an identical termination criterion as the initial test. To enable standardization of relative exercise intensity across volunteers during within-bore exercise, VO_2_ in the final 30 s of each increment was plotted against workload, and a linear regression was used to calculate the workload corresponding to 50% of the supine VO_2_ peak.

Arterialized-venous blood gas analysis was performed during the cardiorespiratory exercise test to ensure the subsequent exercise protocol performed within the MRI scanner was undertaken at an intensity below the ventilatory inflection point such that an increase in CO_2_ partial pressure during exercise did not confound cerebral vascular responses to exercise. A superficial vein on the dorsal surface of a hand was cannulated in a retrograde manner and placed inside a hand warming unit prior to and during exercise to ensure arterialized-venous blood sampling.^25^ Samples were drawn into heparinized syringes and analyzed with a hand-held i-STAT blood gas analyzer (Abbott Point of Care).

### 2.3 MR visit: brain and cardiac function in response to supine exercise, and muscle composition and PCr resynthesis

Participants undertook 2 scan sessions on the same day. First, proton MRI measures were collected on a Philips 3T Ingenia wide-bore scanner. A whole body mDIXON scan was collected to quantify lower limb (calf and thigh) muscle volume (adjusted for body surface area [BSA]) and intra‐muscular fat fraction (FF) (analysis using semi‐automated MATLAB [MathWorks] script). An MPRAGE brain scan estimated gray matter (GM), white matter (WM), and cerebrospinal fluid (CSF) volume with measures corrected for total intracranial volume (Computational Anatomy Toolbox 12 software, Wellcome Department of Cognitive Neurology). Interleaved measures of cardiac output (aortic 2D-QFLOW indexed to BSA to estimate cardiac index [CI]), gray matter cerebral blood flow (gmCBF) (2D‐QFLOW, analyzed using ViewForum, Philips Medical Systems), and brain oxygen extraction fraction (OEF) and gray matter cerebral metabolic rate of oxygen (gmCMRO) (using T(2)‐relaxation‐under‐spin‐tagging analyzed using MATLAB)^26^ were performed at rest, during in‐bore steady‐state supine stepping exercise, and during recovery using an MRI‐compatible Cardiostepper ergometer (Ergospect GmBH). Supine steady‐state isokinetic stepping was performed at a cadence of 70 steps/minute at an intensity of 50% supine VO_2_ peak.

Secondly, calf muscle phosphorus (^31^P) MRS data were collected on a Philips 3T Achieva prior to, during, and following ischemic plantar flexion exercise using an air-braked Trispect MRI compatible ergometer (Ergospect GmbH). Phosphocreatine resynthesis in the medial gastrocnemius muscle following ischemic in‐bore plantar flexion exercise (Trispect, Ergospect GmBH) during occlusion was measured using ^31^P pulse-acquire MRS with 8-second temporal resolution to estimate in vivo muscle mitochondrial function^27^ using jMRUI software.^28^

See the Supplementary Methods for a detailed description of MRI and MRS methodologies.

### 2.4 Statistical analyses

All data were analyzed in IBM SPSS Statistics Version 25. Data were checked for normality using a Shapiro-Wilk test. Between-group comparisons of single independent variables were analyzed by an independent *t*-test, or a Mann-Whitney test for non-parametric data.

Between-group comparisons of cardiac and brain MRI and ^31^P MRS endpoint measures at rest, and during exercise and recovery were achieved using 2-way analysis of variance (ANOVA) with repeated measures. A Bonferroni correction was applied to all pairwise comparisons. Sphericity was assessed by Mauchly’s test of sphericity, if the Greenhouse-Geisser epsilon was ≥0.75, the Huynh-Feldt corrected value was used to correct degrees of freedom, else the Greenhouse-Geisser correction was used. All data are reported as the mean and SEM. Statistical significance was accepted at *P* < .05.

## 3. Results

### 3.1 Participant demographics

**Figure 1.**
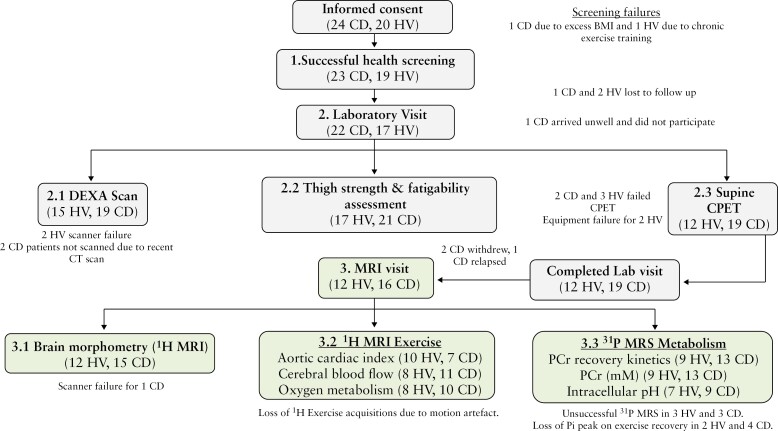
CONSORT diagram detailing volunteer recruitment and progression through the study protocol.

Forty-four volunteers (24 CD, 20 HV) provided written informed consent prior to health screening. Figure 1 details their progression through the study. Twenty-three CD and 19 HV passed health screening, 1 CD failed due to excessive BMI, and 1 HV due to history of chronic exercise training. A further 1 CD and 2 HV passed health screening but were lost to follow-up prior to study visit 1. Of the remaining 39 volunteers (22 CD, 17 HV), 11 (6 CD, 5 HV) were excluded following visit 1, 2 HV were lost to follow-up after equipment failure, 3HV and 2CD failed cardiopulmonary exercise testing (CPET), 2 CD voluntarily withdrew consent following visit 1, 1 CD relapsed after visit 1, and 1 CD arrived unwell on the day of testing and was subsequently lost to follow-up. Twenty-eight volunteers completed the study (16 CD, 12 HV).

CD participants were in clinical remission with an HBI score of 2 ± 1 and had a mean disease duration of 14 ± 3 years. Three participants were being prescribed biological therapies and 4 participants thiopurines at the time of recruitment. Six participants had undergone previous CD-related bowel resections (See Supplementary Table 1). HV and CD groups were matched for age (38 ± 4 vs 42 ± 4 years) and BMI (24 ± 0.9 vs 24.8 ± 1 kg/m^2^) (Table 1).

**Table 1.** Demographic data, fatigue perception measurements, anxiety and depression symptoms, cognitive function assessment.

	CD (*n* = 16, 7 female)	HV (*n* = 12, 9 female)	*P*-value
Age (years)	42 ± 4	38 ± 4	.60
BMI (kg/m^2^)	24.8 ± 1	24.0 ± 0.9	.57
Daily step count	5482 ± 684	8168 ± 1123	.04*
**Fatigue assessment**			
** **MFI—General (0-20)	13.9 ± 1	8.3 ± 0.9	.001*
** **MFI—Physical (0-20)	11.3 ± 1.2	8.4 ± 0.9	.09
** **IBDF S1	8.7 ± 1.4	N/A	N/A
** **IBDF S2	31.5 ± 5.1	N/A	N/A
**Mental health, quality of life, and cognitive function**			
** **HADS (0-21)	10.1 ± 1.4	7.5 ± 1.4	.22
** **CUCQ-32	1.99 ± 0.23	N/A	N/A
** **MoCA (0-30)	26.4 ± 0.6	25.5 ± 0.8	.36
**Inflammatory markers**			
** **TNF-alpha (pg/mL)	21.67 ± 8.83	8.12 ± 2.62	.11
** **IL-1 (pg/mL)	0.39 ± 0.09	0.39 ± 0.13	.63
** **IL-6 (pg/mL)	0.80 ± 0.19	0.21 ± 0.07	.02*
** **CRP (mg/L)	<10	<10	N/A

Unpaired *t*-test; mean ± SEM. *P < .05.

BMI, body mass index; CD, Crohn’s disease; CRP, C-reactive protein; CUCQ-32, Crohn’s and ulcerative colitis questionnaire-32; HADS, Hospital Anxiety and Depression Scale; HV, healthy volunteer; MFI, multidimensional fatigue inventory; MoCA, Montreal Cognitive Assessment.

### 3.2 Calf muscle PCr resynthesis following ischemic plantar flexion exercise

**Figure 2.**
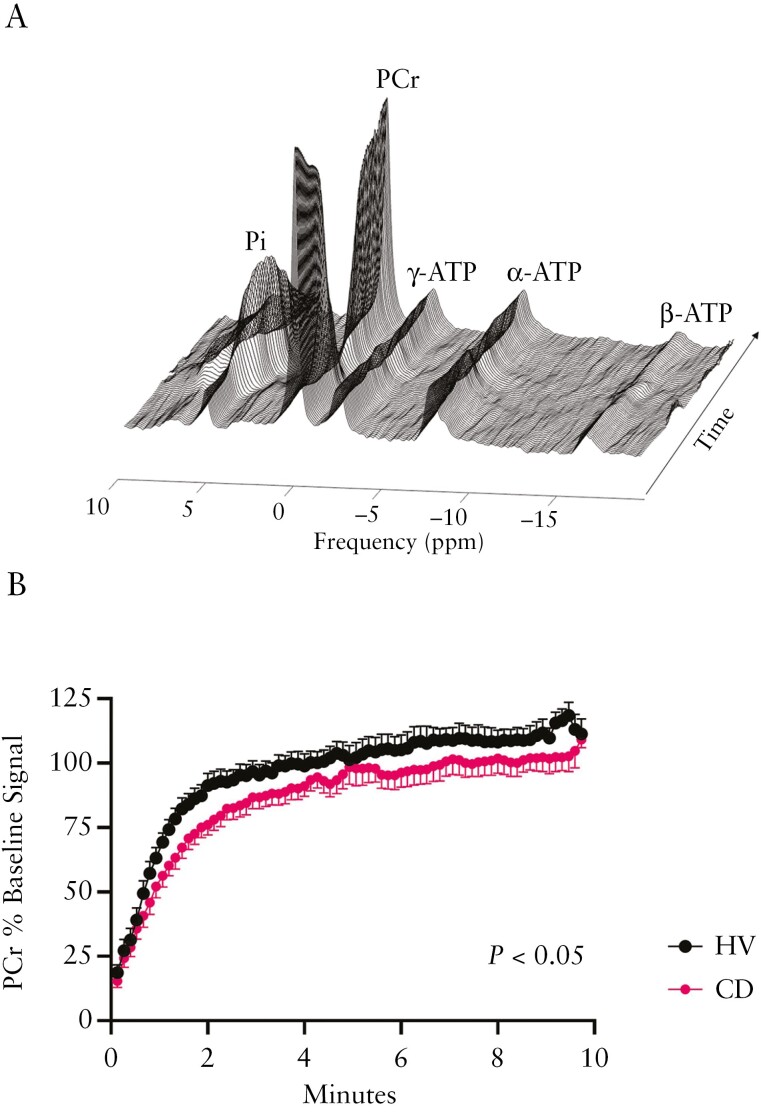
Post-exercise PCr resynthesis after ischemic contraction. (A) Example stacked ^31^P metabolite plot across the exercise task from one volunteer. (B) Mean post-exercise PCr recovery curves as a function of time in 13 CD vs 9 HV. Data presented as mean ± SEM. Error bars are plotted in opposite directions to aid data visualization*P < .05. CD, Crohn’s disease; HV, healthy volunteer; PCr, phosphocreatine.

Example data showing ^31^P MRS tracking of PCr and inorganic phosphate during rest, plantar flexion exercise, and recovery are shown in Figure 2A. Baseline calf muscle PCr concentration was no different between the HV and CD group (Table 2). Ischemic contraction markedly reduced muscle PCr concentration relative to baseline in both groups (*P *< .001, Table 2). The magnitude of end-exercise PCr depletion was well-matched in HV and CD groups equating to PCr degradation of 81 ± 3 vs 85 ± 2% relative to resting PCr concentration (Table 2). After the reinstatement of limb blood flow following ischemic exercise, PCr concentration returned to baseline during recovery in both groups (Table 2). Post-exercise muscle PCr resynthesis was significantly slower in the CD group relative to the HV group (*V*_PCr_ = 17.2 ± 2.0 vs 25.3 ± 2.4 mM·min^−1^, *P* = .02, Figure 2B, Table 2), with data in both groups having a good fit to a mono-exponential recovery function (*r*^2^ = 0.97 ± 0.004 vs 0.98 ± 0.004, respectively).

**Table 2.** ^31^P MRS data across ischemic exercise tasks including calf muscle PCr concentrations, intracellular muscle pH estimations, PCr depletion rates, ^31^P-derived metabolic parameters in healthy volunteers and quiescent Crohn’s disease patients.

	CD (*N* = 13)	HV (*N* = 9)	*P*-value
Baseline calf muscle PCr (mM/L cell water)	26.2 ± 1.3	27.7 ± 1.9	.23
End-exercise calf muscle PCr (mM/L cell water)	3.8 ± 0.6	5.2 ± 1.0	
End-recovery calf muscle PCr (mM/L cell water)	25.3 ± 1.64	28.9 ± 2.11	
Base calf muscle pH	7.20 ± 0.05	7.10 ± 0.04	.32
End-exercise calf muscle pH	6.49 ± 0.05	6.45 ± 0.07	
End-recovery calf muscle pH	7.13 ± 0.07	7.19 ± 0.06	
PCr depletion (%)	84.58 ± 2.42	81.2 ± 3.0	.39
*V*_PCr_ (mM·min^−1^)	17.2 ± 2.0	25.3 ± 2.4	.02*

*P*-values for ^31^P metabolite data across exercise derived from group main effect during 2-way ANOVA. PCr depletion and VPCr from independent *t*-test.

ANOVA, analysis of variance; CD, Crohn’s disease; HV, healthy volunteer; PCr, phosphocreatine; *V*_PCr_, post-exercise PCr resynthesis.

Resting calf muscle intracellular pH at baseline did not differ between groups (Table 2). Ischemic contraction reduced muscle pH at end-exercise (*P* < .001), which was not different between groups (Table 2). Intracellular pH returned to the resting baseline value following recovery in both groups (7.13 ± 0.07 vs 7.19 ± 0.06, main effect of time: *P *< .001, Table 2). The pH kinetics across the exercise task were not different between the HV and CD groups (Table 2).

### 3.3 Cerebral blood flow and oxygenation responses to supine steady-state exercise at 50% VO_2_ peak

**Figure 3.**
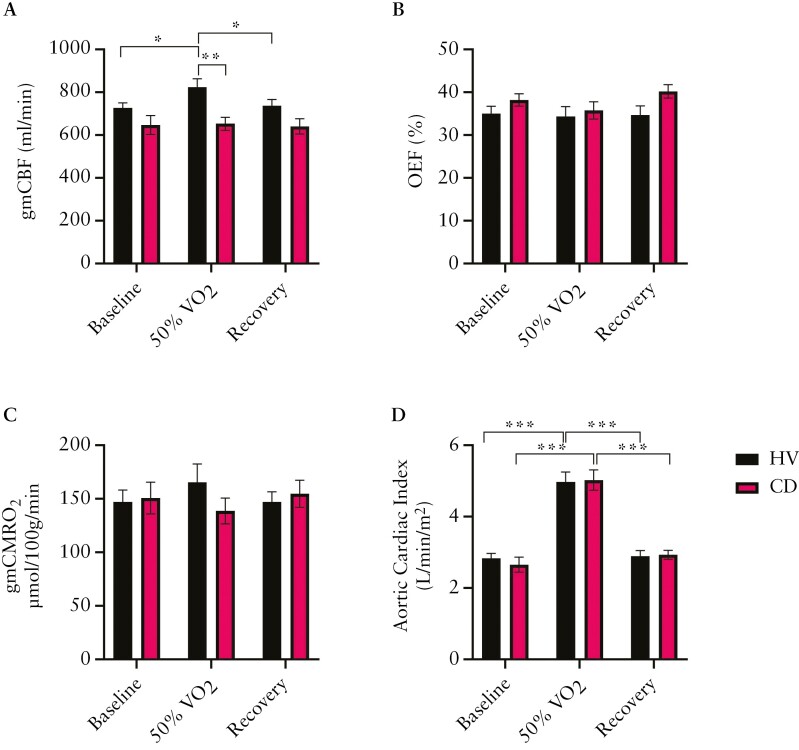
Central vascular and metabolic responses across a sustained low-intensity exercise in HV vs CD. (A) Gray matter corrected cerebral blood flow (gmCBF; 11 CD, 8 HV). (B) Oxygen extraction fraction (OEF; 10 CD, 8 HV). . (C) Gray matter corrected cerebral metabolic rate of oxygen (gmCMRO_2_; 10 CD, 8 HV). (D) Aortic cardiac index (7 CD, 10 HV). Data reported as mean ± SEM. *P < .05, **P < .01, ***P < .001, 2-way mixed design ANOVA. ANOVA, analysis of variance; CD, Crohn’s disease; HV, healthy volunteer.

Gray matter cerebral blood flow was lower in CD than in HV during supine exercise (823 ± 40 vs 653 ± 30 mL/min, *P* = .003; group main effect *P* = .02, Figure 3A), and there was also a main effect of time (*P* = .02, Figure 3A). Relative to baseline, gmCBF in the HV group increased significantly during exercise (727 ± 23 vs 823 ± 40 mL/min, *P* = .027) before returning to resting levels on recovery (737 ± 29 mL/min). However, this response was blunted in the CD group (Interaction effect *P* = .06) where gmCBF did not alter across the task (Figure 3A).

Brain OEF and gmCMRO_2_ were not different between the HV and CD groups, and there was no time effect (Figure 3B and C).

### 3.4 Whole body cardiorespiratory and cardiac responses to supine steady-state exercise at 50% VO_2_ peak

Baseline CI (as measured from the aortic 2D-QFLOW) Figure 3D was no different between HV and CD groups (2.84 ± 0.14 vs 2.66 ± 0.21 L/min). There was a main effect of time for CI across the exercise task (*P* < .001, Figure 3D). Relative to baseline measurements, mean CI increased significantly during exercise in both the HV and CD groups (4.98 ± 0.27 vs 5.03 ± 0.28 L/min, *P *< .001), before decreasing during recovery (*P* < .001) and returning to resting baseline levels (2.89 ± 0.16 vs 2.93 ± 0.13 L/min). Similarly, cardiorespiratory function measures of VE, VCO_2_, VO_2_, and PetCO_2_ were no different between the HV and CD groups (Supplementary Table 2).

### 3.5 Fatigue perception, psychosomatic scores, and daily step count

The CD group self-reported greater general fatigue perception relative to the HV group (13.9 ± 1 vs 8.3 ± 0.9, *P* = .001, Table 1). Physical domain fatigue perception scores were not significantly different between the HV and CD groups (Table 1). CD patients reported IBDF Scale scores of 8.7 ± 1.4 (section 1) and 31.5 ± 5.1 (section 2). Both cognitive function and self-reported anxiety and depression symptoms were comparable between groups (Table 1). Daily step count was less in the CD group compared to the HV group (5482 ± 684 vs 8168 ± 1123, *P *= .04, Table 1).

### 3.6 Serum inflammation measures

In the CD group, all CRP values were <10 mg/L. We observed no difference in serum TNF-alpha and IL-1 between the HV and CD groups (Table 1). Serum IL-6 concentration was significantly greater in the CD group compared to the HV group (0.80 ± 0.19 vs 0.21 ± 0.07 pg/mL, *P *= .02).

### 3.7 Body composition (whole body and regional)

**Figure 4.**
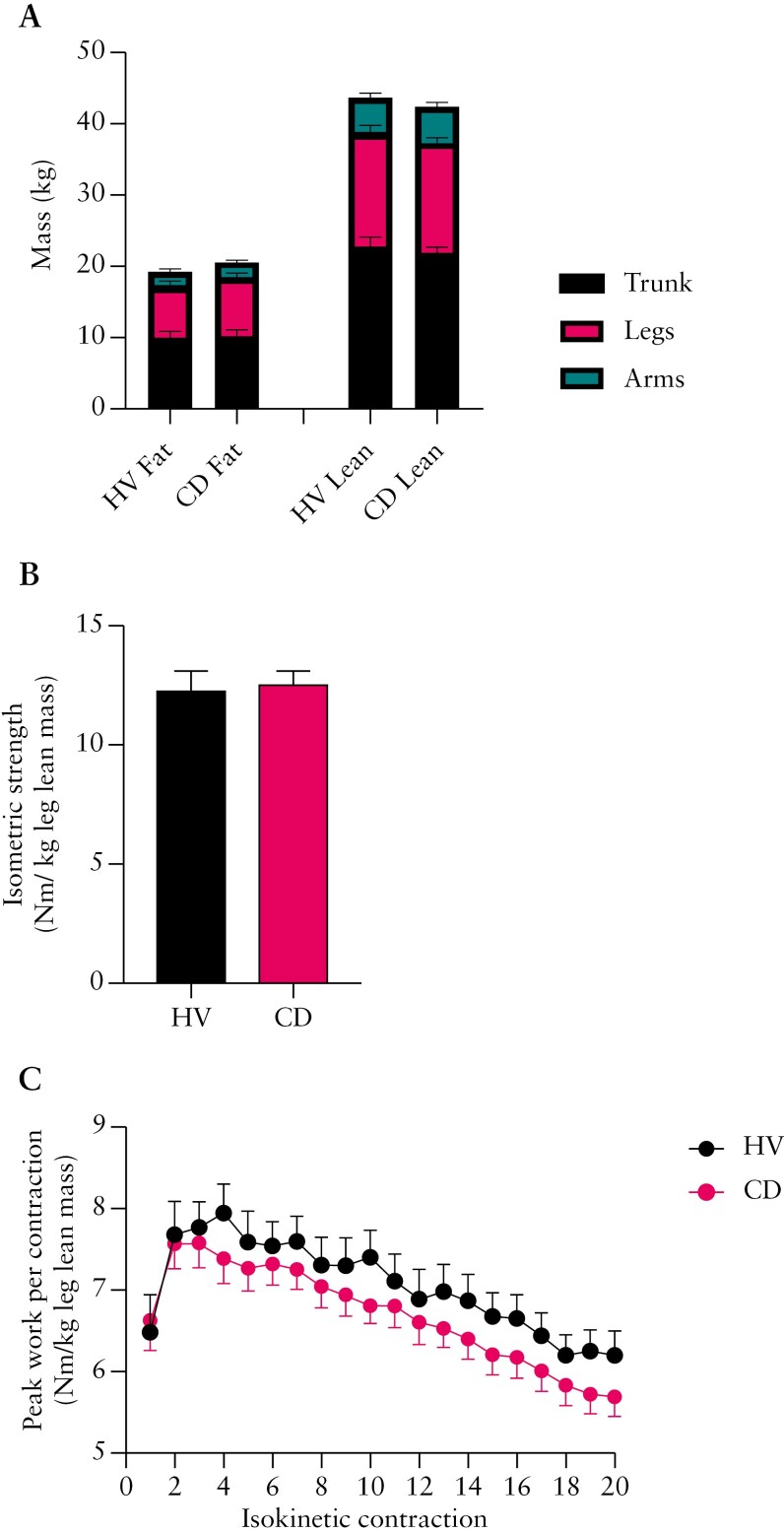
(A) DEXA-estimated regional and whole body composition. (B) Peak isometric thigh strength. (C) Peak isokinetic work across a series of 20 knee extension repetitions. All data presented for 11 CD and 14 HV as mean ± SEM. CD, Crohn’s disease; DEXA, dual-energy X-ray absorptiometry; HV, healthy volunteer.

We could not collect a DEXA scan in 1 HV and 2 CD patients of the 12 HV and 16 CD patients who completed the study (Figure 1). DEXA-estimated regional fat and lean masses were no different between the HV and CD group (Figure 4A). Likewise, both the whole body and appendicular lean mass index, together with bone mineral density, were no different between groups (Table 3). In keeping with this, MRI-derived whole leg and calf muscle volume and intra‐muscular FF were not different between groups (Table 3).

**Table 3.** Body composition and muscle fatigability.

	CD	HV	*P*-value
**DEXA body composition (11HV, 14CD)**			
Whole body lean mass	48.1 ± 2.3	47.8 ± 3.2	.65
Lean mass index	15.8 ± 0.5	16.3 ± 0.8	.65
Appendicular lean mass index	7.1 ± 0.3	7.2 ± 0.4	.86
BMD	1.2	1.2	.52
^**1**^ **H MRI body composition (11HV, 14CD)**			
Whole leg muscle volume	2432 ± 88	2428 ± 81	.97
Whole leg intra‐muscular FF	14 ± 1	12 ± 1	.32
**Muscle fatigability (11HV, 14 CD)**			
Isokinetic work output	131.9 ± 5.7	140.9 ± 5.8	.29
Thigh fatigue index	27.6 ± 2.3	22.8 ± 2.6	.19

DEXA-estimated whole body lean mass (kg), lean mass index (total body lean mass /ht2), and appendicular lean mass index (appendicular lean mass/ht2). ^1^H MRI measurements of whole limb and calf muscle volumes normalized to body surface area (cm3) together with % fat fractions (data presented as FWHM). Muscle fatigability data including isokinetic work output (Nm/kg leg lean mass) and thigh fatigue index (%) during 20 maximal contractions. Unpaired *t-*test; mean ± SEM. *P < .05.

CD, Crohn’s disease; DEXA, dual-energy X-ray absorptiometry; FF, fat fraction; HV, healthy volunteer; ^1^H MRI, proton magnetic resonance imaging.

There was no difference in GM and WM volume between HV and CD participants, but CSF volume was significantly lower in the CD group than in the HV group (Supplementary Table 3).

### 3.8 Leg strength, work output, and exercise fatigue index

Muscle function data normalized to DEXA measurements of leg lean mass are reported for 11 HV and 14 CD participants (Figure 4B and C, Table 3). There was no difference in isometric knee extensor strength (Figure 4B) or isokinetic work output and fatigue index over 20 maximal contractions (Figure 4C, Table 3) when comparing the CD and HV groups.

## 4. Discussion

This study investigated the physiological basis of IBD fatigue by integrating exercise physiology and multiparametric MRI and MRS approaches to achieve unprecedented insight of IBD physiology in quiescent CD who report heightened fatigue perception.

We have shown that the rate of post-exercise muscle PCr resynthesis, a real-time in vivo measure of muscle mitochondrial flux, was significantly slower in the quiescent CD that reported an increased perception of fatigue relative to HV. This difference in muscle PCr resynthesis was not accompanied by between-group differences in body composition (whole body and regional) or measures of CI and muscle strength and work output during exercise, and highlights the presence of greater peripheral muscle deconditioning in CD, which is a trait of other chronic conditions where habitual physical activity levels are diminished.^29^ Of possible importance to the heightened perception of fatigue in CD patients was the finding that GM CBF during exercise was lower compared to HV but was not associated with group differences in brain OEF and gmCMRO_2_.

### 4.1 Peripheral muscle deconditioning

A slowed rate of muscle PCr resynthesis during exercise recovery is a hallmark of muscle deconditioning and is accepted to reflect a decrease in muscle mitochondrial mass and/or reduced mitochondrial function.^30–32^ Of note, this effect is present in aging and chronic diseases with a high prevalence of heightened fatigue perception and premature exercise fatigue. For example, chronic obstructive pulmonary disease (COPD) patients self-report increased fatigue perception,^33^ have a lower muscle mitochondrial density than HV,^34^ and exhibit slowed post-exercise muscle PCr resynthesis,^35^ which is likely underpinned by their physical inactivity.^36^ Importantly, these deficits in muscle mitochondrial function reflected by slowed post-exercise PCr recovery in aging and COPD can be reversed by exercise training intervention,^20,30^ when the exercise regimen is of adequate intensity to stimulate mitochondrial adaptation.^37^ This highlights that intrinsic mitochondrial function in muscle is not impaired by aging or chronic disease and points to a positive role for exercise training intervention as a therapeutic approach for premature exercise fatigue in CD.^15,17,38^ In keeping with this, the CD patients in this study were less active than HV, presenting with a daily step count of 5482 ± 684/day, which is consistent with a sedentary lifestyle index of 5000 steps/day or less^39^ and does not meet recommended daily physical activity guidelines (≥7000 steps/day).^40^ In contrast, the HV group daily step count was 8168 ± 1123 steps/day. Physical inactivity is reported across the lifespan in IBD.^41–43^ This is important as physical activity reduction is associated with reduced mitochondrial function^44^ and other skeletal muscle metabolic deficits. For example, CD patients with blunted muscle hypertrophic signaling were significantly less active relative to patients with normal muscle hypertrophic signaling.^42^ Collectively, these data suggest that physical inactivity is a driver of muscle deconditioning in CD patients with heightened fatigue perception. While the HV group met recommended physical activity guidelines (≥7000 steps/day),^40^ their step count remained relatively low. For example, elderly volunteers (71 ± 4 years) in an aging study classified as having normal activity levels completed 9983 ± 2781 steps per day, while deconditioned, physically impaired elderly participants completed just 6608 ± 1765 steps/day,^45^ which is comparable to the CD cohort in this study. The fact we were able to delineate significant reductions in both step count and muscle mitochondrial function in our CD cohort relative to HV, who themselves demonstrated relatively low levels of recreational physical activity, exemplifies the magnitude of physical inactivity and accompanying deconditioning reported in our CD cohort.

### 4.2 Body composition, muscle strength, and fatigability

Muscle strength has been reported to be less in CD patients than HV and appears to be associated with disease severity.^15,46^ In keeping with some published literature, however, we found that body composition, muscle strength, and exercise fatigue development were not different between quiescent CD patients and HV.^38,47^ At least part of the divergence in these findings is likely to be explained by the assessment of muscle fatigability in IBD being undertaken during sustained isometric contraction,^38,46^ which is very much confounded by muscle venous occlusion that occurs during sustained isometric contraction.^48,49^ Muscle fatigability was assessed in this study by determining total isokinetic work output and fatigue index during 20 repeated maximum voluntary contractions, which is known to recruit quadriceps muscle type I and type II muscle fibers and is not associated with muscle venous occlusion.^50^ While exercise fatigue was clearly evident in both CD patients and HV volunteers, there was no evidence of premature exercise fatigability in CD. Other reasons for the lack of agreement in the literature regarding physical function in CD are also likely explained by differences in disease severity, level of deconditioning, and muscle mass across studies.

### 4.3 Cerebral blood flow

The HV group exhibited an increase in gmCBF from the resting baseline state during exercise, which returned to baseline during recovery. This finding is in keeping with published research demonstrating an increase in cerebral perfusion using transcranial Doppler ultrasound in both young and older volunteers during incremental intensity submaximal exercise,^51^ which is thought to reflect increased neuronal activity and metabolic flux.^52^ However, unlike the HV group, gmCBF did not increase with exercise in the CD group, which was an unexpected novel finding. Brain morphometry measures in the CD group also showed a lower CSF volume than HV, supported by our findings in a larger group,^53^ and a positive association between cerebral blood flow and CSF flow has been reported in human aging. ^54^ Utilizing arterial spin labeling MRI, a decrease in CBF below the resting value has been observed in healthy, young athletes following exhaustive intense exercise. Furthermore, the magnitude of this decline was positively associated with exercise time to fatigue and was not accompanied by a compensatory increase in brain OEF.^55^ It is plausible to suggest, therefore, that the failure of gmCBF to increase during exercise in the CD patients in the present study could have been functionally linked to their heightened perception of fatigue, which warrants further investigation. The precise mechanism for the lack of increase in gmCBF during exercise in CD patients in the present study is unclear, particularly given VCO_2,_ VO_2_, CI, cerebral OEF, and gmCMRO_2_ responses to exercise were no different from HV. Nevertheless, quiescent CD patients with heightened fatigue perception have been reported to show greater cerebral blood flow in the resting state (quantified using ASL-MRI) when compared to healthy age and gender-matched control volunteers.^56^ The present study could not corroborate this observation, but the authors associated the greater cerebral blood flow in CD with concurrent differences in neurochemical and mental health status.

### 4.4 Study limitations

This study involved a relatively small but comprehensively phenotyped cohort of quiescent CD patients. This was important because of the limited understanding of the aetiology of persistent fatigue perception reported in quiescent CD, despite the resolution of disease activity.^4,7^ However, it is acknowledged that this study recruited quiescent IBD patients regardless of the severity of self-reported fatigue perception (i.e. all participants were included), which is in line with previous studies.^38^ Fatigue perception scores used to define clinically significant fatigue perception are without standardization and validated cutoff scores,^2^ and it is therefore important to consider that not all CD patients in the present study self-reported elevated fatigue burden, which may have influenced study outcomes. Future studies should develop validated cutoffs for fatigue assessment scales used in IBD research to enable better stratification of CD patients based on fatigue perception.

Despite ensuring exercise intensity was well controlled during within-bore exercise in the present study, we cannot dispute that the supine cardiorespiratory exercise testing that is essential for MRI-based exercise studies was not as representative of the upright exercise modalities previously employed in CD research.^15,17^

Finally, it was not possible to establish whether the reduced post-exercise PCr resynthesis in CD in the present study that employed in vivo ^31^P MRS and ^1^H MRI was attributable to altered intrinsic mitochondrial function or a reduction in mitochondrial mass compared to HV. However, the latter seems most likely based on published research involving deconditioned older people^30^ and exercise-intolerant patients.^37^

To the best of our knowledge, these data are among the first to comprehensively assess the metabolic and physiological phenotype of quiescent CD relative to age and BMI-matched HV by dovetailing exercise physiology and multiparametric ^1^H MRI and ^31^P MRS approaches. Importantly, we provide evidence of peripheral muscle deconditioning in quiescent CD patients who self-report increased fatigue perception, irrespective of no differences in muscle strength, exercise fatigue, and muscle atrophy from HV. The findings revealing a lower CSF volume, and lack of increase in cerebral blood flow in response to steady-state exercise in CD compared to HV, are novel and warrant further investigation in the context of heightened fatigue perception in CD. Peripheral muscle deconditioning and altered cerebral hemodynamic response to exercise may modulate fatigue perception in IBD, which cannot be explained by deficits in cardiac and muscle function or differences in body composition. These findings give credence to the notion of exercise interventional trials to improve IBD fatigue. Future research efforts should aim to assess the efficacy of exercise training and or step count intervention to target IBD fatigue.

## Supplementary Material

jjae194_suppl_Supplementary_Tables_S1-S3

## Data Availability

The manuscript, data, figures, and tables have not been previously published, and the manuscript is not under consideration elsewhere. Data, analytical methods, and study materials will be made readily available upon discussion with the corresponding author.

## Conference presentation

1. McGing, J. J., Nicholas, R., Serres, S., Gupta, A., Radford, S. J., Bradley, C.,... & Moran, G. (2024). P047 Deconditioning in quiescent Crohn’s Disease patients with heightened fatigue perception. *Journal of Crohn’s and Colitis*, *18*(Supplement_1), i309-i311. (ECCO 2024).

2. McGing, J.J., Nicholas, R., Serres, S., Greenhaff, P., Francis, S., & Moran, G. W. (2022, January). 31P MRS and MRI phenotyping of muscle metabolic quality in IBD fatigue. In *JOURNAL OF CROHNS & COLITIS* (Vol. 16, pp. I171-I172). GREAT CLARENDON ST, OXFORD OX2 6DP, ENGLAND: OXFORD UNIV PRESS. (ECCO 2022).

3. McGing, J.J, Nicholas, R. Serres, S. Greenhaff, PL. Moran, GW & Francis ST. ^*31*^*P MRS and MRI phenotyping of muscle metabolic quality in inflammatory bowel disease fatigue*. Abstract ISMRM 2021 (Virtual).

4. McGing, J.J. Nicholas, R. Serres, S. Greenhaff, P. & Francis, S. (2020). *Assessment of peripheral muscle deconditioning using*^*31*^*P-MRS during high intensity ischeamic plantar flexion exercise.* Accepted Abstract, ISMRM, Sydney 2020.
