# Supplementary material for: Deconditioning in quiescent Crohn’s disease patients with heightened fatigue perception
Source: J Crohns Colitis. 2025 Jan 9;19(1):jjae194. doi: 10.1093/ecco-jcc/jjae194 (PMC11786219; doi:10.1093/ecco-jcc/jjae194)
Supplement: jjae194_suppl_Supplementary_Tables_S1-S3 [file jjae194_suppl_supplementary_tables_s1-s3.docx]

**Supplementary Material**

| **ID** | **Disease phase** | **Sex** | **Age (years)** | **BMI (kg/m^2^)** | **Medication** | **Surgery** | **Disease duration** | **HBI** |
| --- | --- | --- | --- | --- | --- | --- | --- | --- |
| 3 | R | F | 31 | 26.8 | Amitriptyline, Infliximab. | Ileocecal Resection. | 17 | 5 |
| 5 | R | F | 33 | 33 | Vedolizumab. | Hemicolectomy. | 9 | 4 |
| 6 | R | M | 52 | 31.2 | Lansoprazole. | Resection. | 18 | 2 |
| 8 | R | M | 26 | 19.7 | Loperamide, Mebeverine, Mirtazipin, ferrous sulphate. | 11 Exploratory / abscess related peri anal surgeries & loop ileostomy. | 4 | 1 |
| 11 | R | M | 54 | 23.5 | Mesalazine Mebeverine, Sertaline | Perianal abscess, small bowel resection. | 29 | 0 |
| 12 | R | F | 44 | 22.6 | Mesalazine |  | 17 | 1 |
| 14 | R | F | 53 | 21 |  |  | 3 | 0 |
| 21 | R | M | 23 | 22 | Azathioprine, ferrous fumarate. |  | 8 | 2 |
| 24 | R | M | 62 | 26.7 |  |  | 12 | 7 |
| 25 | R | F | 25 | 23 | Infliximab |  | 4 | 0 |
| 32 | R | F | 54 | 23.5 | Mercaptopurine | Hemicolectomy. | 28 | 2 |
| 33 | R | M | 26 | 22 |  |  | 13 | 1 |
| 35 | R | F | 41 | 21.5 | Mercaptopurine |  | 3 | 3 |
| 37 | R | F | 26 | 31.4 |  |  | 4 | 1 |
| 38 | R | F | 58 | 22.4 |  |  | 40 | 2 |
| 40 | R | M | 60 | 26.3 | Mesalizine, Azathioprine |  | 18 | 0 |

***Supplementary Table 1****. Clinical parameters for CD patients.*

|  | CD (N=10) | HV (N=10) | *P* - value |
| --- | --- | --- | --- |
| VE (L/min) | 23 ± 2 | 25 ± 2 | 0.31 |
| VCO_2_ (mL/kg/min) | 11 ± 1 | 13 ± 1 | 0.17 |
| VO_2_ (mL/kg/min) | 12 ± 1 | 13 ± 1 | 0.35 |
| petCO_2_ (mmHg) | 39 ± 1 | 42 ± 1 | 0.07 |

**Supplementary Table 2. Interpolated cardiorespiratory parameters at 50% supine VO_2_ peak for volunteers that completed the within-bore ^1^H MRI exercise task.** Minute ventilation (VE), Expired CO_2_ volume (VCO_2_) oxygen uptake (VO_2_) and Partial pressure of end-tidal CO_2_ (petCO_2_). Data presented as mean ± SEM

| **MRI Brain morphometry** | | | |
| --- | --- | --- | --- |
|  | CD (15) | HV (12) |  |
| Grey matter volume (cm^3^) | 639± 18 | 624 ± 27 | 0.64 |
| White matter volume (cm^3^) | 534 ± 10 | 512± 17 | 0.26 |
| Cerebrospinal fluid (mL) | 237 ± 10 | 281 ± 15 | 0.02* |

***Supplementary Table 3. ^1^H MRI brain morphometry in CD and HV.*** *Grey matter, white matter and cerebrospinal fluid (CSF) volume, measured normalised by total intracranial volume (TIV), a significant difference was seen in CSF volume.*

**Supplemental Methods**

## **MR data acquisition**

Participants undertook two MR scan sessions on the same day.

In the first scan session, ^1^H MRI measures were collected on a Philips 3T Ingenia wide-bore scanner. A dStream HeadSpine coil and two anterior body coils were used to collect a MultiStation 6-stack whole body mDIXON scan from which to quantify lower limb composition (muscle volume and muscle fat fraction) (FOV 400 x 492 x 231 mm^3^, resolution 2.5 x 2.5 x 6 mm^3^, 77 slices, flip angle (FA) 3˚, 6 echoes, shortest TR/TE1/ΔTE2). An MPRAGE brain scan (256 x 256 x 160 matrix, FA 8°, TR/TE 8.3/3.8 ms, SENSE factor 2) was collected for morphometry measurements. Participants were then secured into a Cardio Step ergometer, fixing the knee angle with a goniometer to ensure consistency with the physiology laboratory exercise test. A dStream Head Neck Spine coil and FlexCoverage posterior coil integrated in the scanner bed were then used to collect brain and cardiac scans at rest and during supine steady-state exercise. A phase contrast (PC) MRI scan through the carotid and basilar arteries (cardiac triggered, FOV 280 x 77 x 6 mm^3^, resolution 0.75 x 0.75 x 6 mm^3^, FA 25°, TE/TR = 6.5/15 ms, SENSE factor 3, V_ENC_ 100 cm/s, NSA = 2) was collected to measure global cerebral blood flow (CBF). Brain fractional oxygen extraction fraction (OEF) was determined using a T_2_ Relaxation Under Spin Tagging (TRUST) sequence (FOV 230 x 230 mm^2^, spatial resolution 3.44 x 3.44 x 5 mm^3^, TI = 1022 ms, and TR = 3000 ms per label/control pair. The scheme used a Flow Alternating Inversion Recovery (FAIR) labelling scheme with selective/nonselective thickness of 25/300 mm followed by collection of data at four eTEs were using in a Carr-Purcell-Meiboom-Gill scheme at 1, 40, 80, 160 ms corresponding to 0, 4, 8 and 16 refocussing pulses in the T_2_ preparation with an interval τ_cpmg_ = 10 ms. This resulted in a measure of T_2_ in sagittal sinus and thus Y_v_, and together with CBF this was used to estimate the cerebral metabolic rate of oxygen (gmCMRO_2_). A cardiac triggered free breathing PC-MRI sequence through the aorta was used to measure cardiac index (CI). The supine in-bore exercise was performed at an intensity equivalent to 50% supine VO_2_ peak for ~ 15 minutes and brain PC-MRI and TRUST and cardiac PC-MRI date were collected after 3 min to ensure steady-state conditions were achieved. Step frequency and power output were continuously monitored via a monitor in the console room and a metronome was played through headphones at a frequency of 70 steps per min. Upon completion of the exercise, recovery brain PC-MRI and TRUST and cardiac PC-MRI were immediately acquired for ~ 15 minutes.

In the second scan session, calf muscle phosphorus (^31^P) MRS data were collected (Philips 3T Achieva with multi-nuclear capability) of the dominant limb prior to and following ischaemic plantar flexion exercise was collected using an air braked Trispect MRI compatible ergometer (Ergospect GmbH, Innsbruck, Austria). First, isometric strength was determined from the peak force generated during three maximum voluntary plantar flexion manoeuvres. Participants were instructed to push maximally against an immovable resistance for 3 s followed by 60 s recovery. Following a 5 min rest period, a high-resolution axial calf mDIXON scan (FOV 208 x 189 x 250 mm^3^, matrix 140 x 126 x 50, FA 10°, TR/TE1/TE2 3.4/1.1/2.2) was acquired and reconstructed in the sagittal and coronal planes A 16 min non-localized pulse-acquire ^31^P-MR assessment followed (TR 4s, TE 0.10 ms, spectral bandwidth 3000 Hz, samples 4096). ^31^P spectra were collected with the ^31^P coil secured over the medial gastrocnemius muscle under resting conditions for 1 min, a cuff around the thigh was then inflated to 250 mmHg to occlude lower limb blood flow, which was maintained for 2 min prior to ischemic plantar flexion exercise commencing at an intensity of 50% MVC until contractile failure (to deplete muscle phosphocreatine content). Post-exercise ischemia was maintained for 30 s to allow spectral averaging, after which lower limb blood flow was reinstated. ^31^P MRS data collection continued throughout the recovery period (~ 10 min, Figure. 2B).

**MRI Data Analysis**

*Muscle volume and fat fraction:* Muscle volume and intra‐muscular fat fraction (FF) was estimated from the mDIXON images using a FSL and MATLAB pipeline. First the water (W) image was fed into FSL’s FAST function, which was set to segment three tissue types. The image was then cleaned by removing the skin on the perimeter of the image, using a Euclidian distance transform for extraneous objects, and using the fat fraction map to eliminate any instances where the fat fraction was above 60%. From this the muscle volume was then estimated and the mean intra‐muscular FF within this computed.

*Cardiac output and cerebral blood flow:* Cardiac triggered (PC) MRI were processed using View forum software (Philips Medical Systems, Netherlands). An ROI was drawn around the walls of the ascending aorta, internal carotid and basilar arteries using the magnitude image. The ROIs were then propagated across all phases in the cardiac cycle and manually edited to optimise fitting. Flux values were used to compute cardiac output and global cerebral blood flow (CBF). CBF was normalised to grey matter volume (gmCBF) derived from MPRAGE to account for structural atrophy.

*Brain oxygen extraction fraction:* TRUST data were analysed using in-house MATLAB code according to previously described methods^1^. Venous blood signal in the superior sagittal sinus (SSS) was computed from the pairwise subtraction of label and control images to obtain difference images for each eTE. In the resultant difference images, an ROI of four voxels with the highest difference signal created a mask for spatial averaging of the signal. Blood T_2_ relaxation was then calculated within the superior sagittal sinus (SSS) ROI by fitting to the mono-exponential signal decay. The blood T_2_ relaxation time in the SSS was then converted into venous oxygenation, Yv, using a calibration plot^2^.

Arterial oxygenation, $Y_{a}$ was calculated as described by Peng et al^3^.

|  | $Y_{a}=99.77-0.036 \times age-1.235 \times sex+0.021 \times age \times sex$ | (1) |
| --- | --- | --- |

where *age* is in years and *sex* is defined as 0 for females and 1 for males^4^.

Oxygen extraction fraction was calculated by

|  | $OEF=Y_{a}-Y_{v}$ | (2) |
| --- | --- | --- |

gCMRO_2_ was calculated as described by Liu et al^5^

|  | $gCMRO_{2}=gCBF \times\left( Y_{a}-Y_{v} \right)\times C_{h}$ | (3) |
| --- | --- | --- |

where *gCMRO2* and *gCBF* are global cerebral metabolic rate of oxygen and global cerebral blood flow (gCBF) and $C_{h}$ is the capacity of blood to transport oxygen. $C_{h}$ values were calculated for each subject from their haemoglobin concentrations. $C_{h}$ was then adjusted for age assuming a decline rate of 0.0079 mmol/L per year^6^.

gmCMRO_2_ was then corrected for brain atrophy to account for any disease or age-related loss of grey and white matter between groups^3^.

|  | $gmCMRO_{2} = \frac{tCMRO_{2}}{V_{gm}+rV_{wm}}\times\frac{1}{\rho}$ | (4) |
| --- | --- | --- |

where *gmCMRO_2_* is the *CMRO_2_* normalised to grey matter volume, $V_{\mathrm{gm}}$ and $V_{\mathrm{wm}}$ are the grey and white matter volumes, *r* is the ratio between white and grey matter volume and $\rho$ is a constant relating to the mass density of tissue (1.06 g/ml)^7^.

*Calf muscle PCr recovery kinetics:*

^31^P spectra were collected at rest for 1 min, a cuff around the thigh was then inflated to 250 mmHg to occlude lower limb blood flow, which was maintained for 2 min prior to ischemic plantar flexion exercise commencing at an intensity of 50% MVC until contractile failure (to deplete muscle phosphocreatine content), A 30 s period was collected post contractile failure, after which lower limb blood flow was reinstated and ^31^P MRS data collected throughout a ~10 min recovery period. PCr kinetics were fit to a mono-exponential function, and baseline and end-exercise PCr concentration and pH computed. Non-localized pulse-acquire ^31^P-MR spectra were analysed using jMRUI Beta 6.0^8^. ^31^P-MR spectra were apodized to 10 Hz with Lorentzian fitting. ^31^P spectra peaks including inorganic phosphate (Pi) PCr, and ATP subunits $\left( \gamma-ATP, \alpha-ATP, ß-ATP \right)$ were fit using the AMARES function with prior knowledge. The exercise kinetics for PCr was expressed relative to baseline signal amplitude. Cytosolic pH was calculated using the chemical shift difference $(\delta)$ between Pi and PCr peaks,

|  | $pH = pK + log\left( \frac{\delta1-\delta0}{\delta0-\delta2} \right)$ | (5) |
| --- | --- | --- |

where p*K* = 6.75, $\delta$1 = 3.27, $\delta$2 = 5.63.

The PCr recovery curve was fit to a mono-exponential function in GraphPad prism.

|  | (PCr(*t*) = PCr_initial_+(PCrend – PCr_initial_)(1-exp(-k*t*)) | (6) |
| --- | --- | --- |

where t is the time from the start of recovery, PCr_initial_ and PCr_end_ is the PCr content at the initial and end of recovery phases.

Post exercise PCr resynthesis rate was estimated as

|  | $VPCr = k\mathrm{PCr}\times\Delta[PCr]$ | (7) |
| --- | --- | --- |

where *k*_PCr_ is the rate constant of PCr resynthesis and $\Delta[PCr]$ is the end recovery PCr concentration minus the end-exercise PCr concentration in mM/L cell water^9,10^.

**References**

1. Lu H, Ge Y. Quantitative evaluation of oxygenation in venous vessels using t2-relaxation-under-spin-tagging mri. *Magnetic Resonance in Medicine* 2008;**60**:357-63.

2. Zhao JM, Clingman CS, Närväinen MJ, Kauppinen RA, Van Zijl PC. Oxygenation and hematocrit dependence of transverse relaxation rates of blood at 3T. *Magnetic Resonance in Medicine: An Official Journal of the International Society for Magnetic Resonance in Medicine* 2007;**58**:592-7.

3. Peng S-L, Dumas JA, Park DC*, et al.* Age-related increase of resting metabolic rate in the human brain. *Neuroimage* 2014;**98**:176-83.

4. Lu H, Xu F, Rodrigue KM*, et al.* Alterations in cerebral metabolic rate and blood supply across the adult lifespan. *Cerebral cortex* 2011;**21**:1426-34.

5. Liu P, Xu F, Lu H. Test–retest reproducibility of a rapid method to measure brain oxygen metabolism. *Magnetic resonance in medicine* 2013;**69**:675-81.

6. Aanerud J, Borghammer P, Chakravarty MM*, et al.* Brain energy metabolism and blood flow differences in healthy aging. *Journal of Cerebral Blood Flow & Metabolism* 2012;**32**:1177-87.

7. Herscovitch P, Raichle ME. What is the correct value for the brain-blood partition coefficient for water? *Journal of Cerebral Blood Flow & Metabolism* 1985;**5**:65-9.

8. Stefan D, Di Cesare F, Andrasescu A*, et al.* Quantitation of magnetic resonance spectroscopy signals: The jmrui software package. *Measurement Science and Technology* 2009;**20**:104035.

9. Kemp GJ, Ahmad RE, Nicolay K, Prompers JJ. Quantification of skeletal muscle mitochondrial function by 31p magnetic resonance spectroscopy techniques: A quantitative review. *Acta Physiologica* 2015;**213**:107-44.

10. Heskamp L, Lebbink F, van Uden MJ*, et al.* Post‐exercise intramuscular O_2_ supply is tightly coupled with a higher proximal‐to‐distal atp synthesis rate in human tibialis anterior. *The Journal of physiology* 2021;**599**:1533-50.
